# Comprehensive Insights into Ochratoxin A: Occurrence, Analysis, and Control Strategies

**DOI:** 10.3390/foods13081184

**Published:** 2024-04-12

**Authors:** Yamina Ben Miri, Amina Benabdallah, Imene Chentir, Djamel Djenane, Andrea Luvisi, Luigi De Bellis

**Affiliations:** 1 Department of Biochemistry and Microbiology, Faculty of Sciences, Mohamed Boudiaf University, BP 166, M’sila 28000, Algeria; yamina.benmiri@univ-msila.dz; 2 Laboratory on Biodiversity and Ecosystem Pollution, Faculty of Life and Nature Sciences, University Chadli Bendjedid, El-Tarf 36000, Algeria; benabdallah-amina@univ-eltarf.dz; 3 Laboratory of Food, Processing, Control and Agri-Resources Valorization, Higher School of Food Science and Agri-Food Industry, Algiers 16200, Algeria; i.chentir@essaia.dz; 4 Food Quality and Safety Research Laboratory, Department of Food Sciences, Mouloud Mammeri University, BP 17, Tizi-Ouzou 15000, Algeria; djenane6@yahoo.es; 5 Dipartimento di Scienze e Tecnologie Biologiche ed Ambientali, Università del Salento Palazzina A—Centro Ecotekne via Prov, le Lecce Monteroni, 73100 Lecce, Italy; andrea.luvisi@unisalento.it

**Keywords:** ochratoxin A, toxin, contamination, health complication, control

## Abstract

Ochratoxin A (OTA) is a toxic mycotoxin produced by some mold species from genera *Penicillium* and *Aspergillus*. OTA has been detected in cereals, cereal-derived products, dried fruits, wine, grape juice, beer, tea, coffee, cocoa, nuts, spices, licorice, processed meat, cheese, and other foods. OTA can induce a wide range of health effects attributable to its toxicological properties, including teratogenicity, immunotoxicity, carcinogenicity, genotoxicity, neurotoxicity, and hepatotoxicity. OTA is not only toxic to humans but also harmful to livestock like cows, goats, and poultry. This is why the European Union and various countries regulate the maximum permitted levels of OTA in foods. This review intends to summarize all the main aspects concerning OTA, starting from the chemical structure and fungi that produce it, its presence in food, its toxicity, and methods of analysis, as well as control strategies, including both fungal development and methods of inactivation of the molecule. Finally, the review provides some ideas for future approaches aimed at reducing the OTA levels in foods.

## 1. Introduction

Mycotoxins, which are naturally synthesized by fungi, can induce toxic responses if contaminated food is consumed by animals or humans. These compounds have gained global attention due to their remarkable economic implications, which are linked to their impact on human health, animal productivity, and food international trade [1,2].

Notably, the presence and proliferation of mycotoxigenic fungi species can exert a considerable influence on the quality and security of food resources [3].

Mycotoxins have been found in a wide range of products, spanning from raw ingredients to processed foods, including staples like bread, flour, pasta, and fruit juice [4]. While numerous mycotoxins have been identified, about 20 families cause issues in human and animal food. Among these, six families frequently occur in food commodities, including aflatoxins (AFs), ochratoxins (OTs), fumonisins (FBs), deoxynivalenol (DON), zearalenone (ZEA), and patulin [5,6]. Furthermore, their thermal stability often exceeds that of the fungi that produce them, making the elimination of mycotoxins difficult or impossible [7,8]. Exposure to mycotoxins can lead to both acute and chronic toxicities, ranging from death to detrimental impacts on various systems, including the central nervous system, cardiovascular system, respiratory system, digestive system, urinary system, and intestinal fibrosis [9]. Of note is their ability to modulate the immune system, thereby reducing resistance to infections, an effect that is now widely recognized as highly significant [10]. Supplementary Table S1 presents a summary of mycotoxins, the producing species, the contaminated foods, and the main effects on humans [11,12,13,14].

AFs, which are produced by *Aspergillus* species, are notorious for their potent carcinogenicity, and commonly contaminated crops are peanuts, corn, and tree nuts [15,16]. FBs, which are produced by *Fusarium* species, are common in maize and maize-based products [17]. DON and ZEA, which are also produced by *Fusarium* species, are found in cereals and grains [18,19]. Patulin, which is produced by *Penicillium* and *Aspergillus* species, have been detected in apple products and fruit juices [20,21]. Ochratoxins, mainly ochratoxin A (OTA), which is the subject of this review, are produced by *Aspergillus* and *Penicillium* species and can be found in foodstuffs, including cereals, coffee, cheese, and wine [22,23]. A single food product can be susceptible to multiple contaminations. Toxigenic fungi have the capacity to produce several mycotoxins, and the fungi of diverse species can synthesize identical mycotoxins. Additionally, the presence of a mycotoxin-producing fungus does not consistently correspond to the presence of mycotoxins in food products. Thus, humans and animals are often exposed not to a singular mycotoxin but frequently to a mixture of mycotoxins [24,25].

This review aims to cover all primary issues related to OTA, the best-known toxin among ochratoxins because of its widespread occurrence and good stability of the molecule. It contaminates a wide range of agricultural products, including cereal grains (wheat and corn), fruit juices, wine, tea, beer, and coffee [23,26,27,28,29,30]. Because these products are the basis of the human diet, it is virtually impossible to avoid consuming OTA, and in turn it originates toxic effects on humans such as carcinogenicity, immunotoxicity, neurotoxicity, hepatotoxicity, teratogenicity, and genotoxicity [31]. The same report indicates that cereals are the main source of OTA contamination, followed by wine, grape juice, coffee (roasted), and pork. As a consequence, mainly to prevent damage to human health, many countries have established permitted levels of OTA in foodstuffs, e.g., in the European Union, the maximum allowable value in cereals is 3 µg/kg [32]. This review describes the OTA chemical structure and fungi responsible for its production, occurrence in food, analytical methods, and strategies for control (involving both the management of fungal growth and approaches for the deactivation of the toxin). Furthermore, it concludes by presenting suggestions for future approaches aimed at mitigating the OTA issue.

## 2. Ochratoxin A (OTA): Chemical Structure, Fungal Producers, and Conditions for Formation in Commodities

OTA is produced by different fungi, primarily belonging to the genera *Aspergillus*, *Penicillium*, and *Fusarium*, including species such as *A. ochraceus*, *A. carbonarius*, *A. niger*, and *P. verrucosum* [33]. The principal fungi capable of toxin production are indicated in Table 1. Contamination generally arises from suboptimal agricultural methods, incomplete or too slow dehydration processes, and inadequate storage practices [6], while the coexistence of mycotoxigenic fungi and mycotoxins in seafood has recently become a source of serious global concern [34].

**Figure 1:**
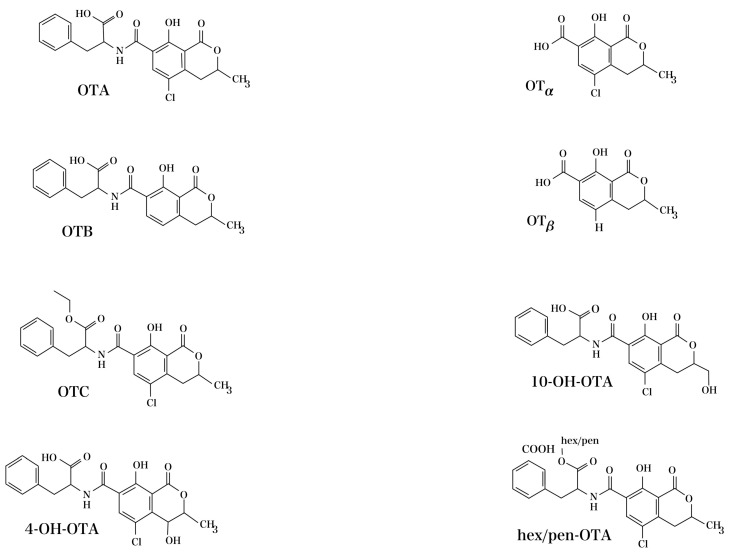
Chemical structure of OTA and its metabolites.

Originally discovered by South African scientists in 1965 [45], the chemical identity of OTA, which is formally known as L-phenylalanine, N-[[(3R)-5-chloro-3,4-dihydro-8-hydroxy-3-methyl-1-oxo-1H-2-benzopyran-7-yl]carbonyl]-, molecular formula C_20_H_18_C_l_NO_6_, reveals an intricate structure. OTA is a member of the dihydrocoumarins. It is made up of an isocoumarin group that has been substituted (7-carboxy-5-chloro-8-hydroxy-3,4-dihydro-3R methyl isocoumarin) and is joined to L-ß-phenylalanine via an amide bond [46,47]. Figure 1 shows the chemical structure of OTA and its intermediary compounds, including ochratoxin B (OTB), ochratoxin alpha (OTα), and ochratoxin beta (OTβ).

Pure OTA possesses a molar mass of 403.8 g/mol, and it appears as a white solid, with a melting point of 90 °C when crystallized from benzene; however the melting point climbs to 169 °C if OTA is crystallized from xylene [46]. OTA has a pKa of 7.1, making it a weak acid. OTA exhibits solubility in polar organic solvents under neutral and acidic conditions and has low solubility in aqueous solutions. However, when the pH is basic, it dissolves in alkaline/sodium bicarbonate-containing aqueous solutions. When exposed to ultraviolet (UV) radiation, OTA exhibits extraordinary fluorescence, generating a green fluorescence in an acidic environment and a blue fluorescence in an alkaline environment [46]. The OTA detection and analysis techniques are built on top of this peculiar fluorescence [47]. Generally stable, OTA can be kept in a refrigerator when dissolved in ethanol or methanol, and it is resistant to radiation and temperature [48].

The OTA-producing fungi are influenced by various factors, such as temperature, pH, moisture content, and water activity (a_w_). The a_w_ is a critical factor for mold germination and growth on nutrient-rich substrates; for example, *A. ochraceus* produces very little OTA at a_w_ 0.80 on green coffee but a considerable amount of OTA at a_w_ value of 0.95: 7.2 mg/kg [49]. Temperature is another essential factor, with optimal ranges for OTA production being 25–30 °C for *A. ochraceus*, 10–20 °C for *A. carbonarius*, and 20–25 °C for *A. niger* [50,51,52]. In addition, the production of OTA by different fungal species is influenced by specific climate conditions. In South America, South Asia, and Africa, which are characterized by hot and relatively dry climates, *Aspergillus* species are the primary producers of OTA on maize [53]. Conversely, in temperate countries like the United States, Canada, and Europe, the *Penicillium* genus is the dominant OTA producer on cereals [54]. Additionally, the development of *P. verrucosum* and OTA formation varies in different grains, such as wheat, depending on moisture content and temperature, starting at 16% humidity [55] and rising with increasing humidity in the range of 10 to 28 °C [56]. Gas composition also influences fungal growth and OTA production in various food items, including dried fruit, alcoholic beverages, and coffee. On wheat grains, *P. verrucosum* growth and OTA production are inhibited by 50% CO_2_ [57]. OTA is mainly produced on moderately acidic foodstuffs, and pH does not have specific effects for *A. ochraceus* and *A. carbonarius* [58], even though a pH between 5.5 and 6.5 was optimal for the growth and OTA production of *P. verrucosum* and *P. nordicum* [59], and a pH of 5.35 was optimal for *A. carbonarius* and *A. niger* OTA production [60].

## 3. OTA Biosynthetic Pathway and Regulatory Mechanisms

**Figure 2:**
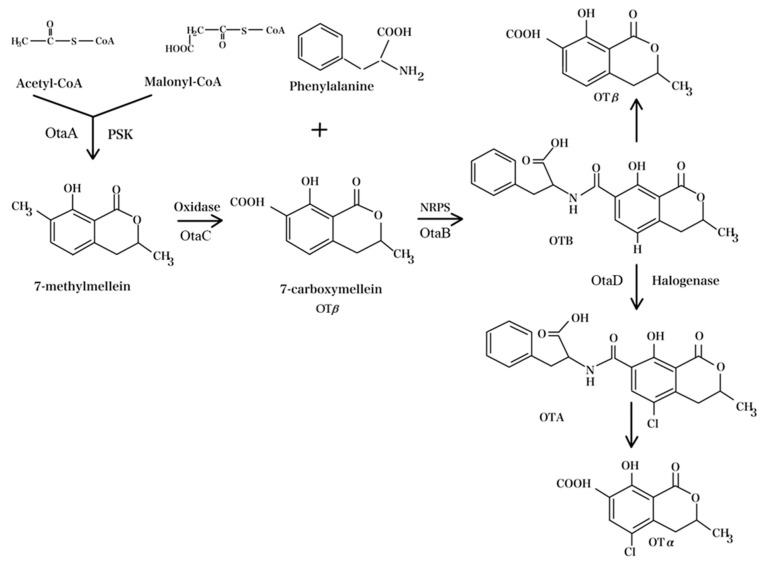
Proposed OTA biosynthetic pathway, including intermediate metabolites and catalytic enzymes (redraw after [68]).

A key point of the OTA biosynthetic pathway was the identification and sequencing of the gene responsible for encoding a halogenase/chloroperoxidase [61]. A mutation in this gene resulted in an increased OTB level and an undetectable amount of OTA in *A. carbonarius*, indicating that the addition of a chlorine atom by halogenase represents the final biochemical step [62]. Genes encoding OTA biosynthetic enzymes were previously identified in *A. carbonarius* as multimodule enzymes like polyketide synthases (*PKS*) and non-ribosomal peptide synthases (*NRPS*) [63]. Chloroperoxidase’s role in introducing chlorine into OTA was identified in ochratoxigenic strains. The polyketide enzyme family includes domains *like KS* (β-ketosynthase), *AT* (acyltransferase), *DH* (dehydratase), and *C-Met* (C-methyltransferase), which are responsible for adding the methyl group to the OTA molecule, with *ER* (enoyl reductase), *KR* (ketoreductase), and *ACP* (acyl-CoA precursor carrier) domains located in the C-terminus. NRPS in fungi extends amino acid chains via modules reflecting the final product’s amino acid count and sequence [64,65]. The involvement of chloroperoxidase in the OTA pathway is supported by its chemical structure, with the chloroperoxidase genes, encoding the enzymes that add a chlorine atom at the C5 position, identified in *A. carbonarius* (*AcOTAhal*) and *P. nordicum (otachlPN)* [62,66,67]. In a study by Wang et al. [68], the OTA biosynthesis process (as depicted in Figure 2) was proposed to initiate with the enzyme OtaA, a PKS, which synthesizes 7-methylmellein using acetyl coenzyme A (1 acetyl-CoA) and malonyl-CoA. Subsequently, OtaC oxidizes 7-methylmellein into OTβ (7-carboxymellein), while OtaB, an NRPS, catalyzes the formation of an amide bond between OTβ and L-β-phenylalanine, resulting in OTB. Finally, OtaD, a halogenase, chlorinates OTB, resulting in the formation of OTA.

Hormonal signaling pathways and transcription factors have been identified as regulators of OTA biosynthesis. One significant regulatory complex is the “VelB/VeA/LaeA” velvet complex, which controls fungal development and secondary metabolite production in fungi also interacting with light receptors [69,70], particularly *Aspergillus* species [71,72]. In addition, Wang et al. [68] discovered two regulators of OTA biosynthesis *in A. ochraceus*, OtaR1, and OtaR2. Specifically, a mutation in *OtaR1* determines a low-level expression of the *OtaABCD* genes, while the second regulator, OtaR2, modulates the expression of the *OtaA*, *OtaB*, and *OtaD* genes. The authors asserted that their comprehensive research has clarified uncertainties surrounding the OTA biosynthetic mechanism. Cell redox balance has also been implicated, as demonstrated by the disruption of the *Aoyap1* gene in *A. ochraceus*, which, downregulating the antioxidant response of the cell, triggers OTA biosynthesis [73]. Fungi prefer easily metabolizable carbon sources, reducing the synthesis of enzymes related to other carbon sources, to increase OTA production growing in the presence of sugars, particularly fructose [36,74]. Osmotic stress, particularly in the case of high NaCl levels, influences OTA production differently among fungi. *Penicillium* species adapt to NaCl-rich environments by producing OTA as a mechanism to maintain cellular homeostasis. However, *A. carbonarius* lacks this adaptation and has limited OTA biosynthesis under osmotic stress caused by NaCl. The high osmolarity glycerol (HOG) signaling cascade, which is activated by osmotic stress, drives OTA production in NaCl-rich environments, but *A. carbonarius* appears to decouple OTA biosynthesis from this mechanism [75,76]. Hence, there is a need for further investigation to clarify the mechanisms by which OTA biosynthesis regulation responds to environmental signals [68].

## 4. Ochratoxin A Occurrence in Food

OTA has been identified in various agricultural products, including cereals and their byproducts, coffee, cocoa beans and chocolate, dried fruits, nuts, chili sauce, spices, ham, pork meat and salami, meat and meat products, poultry, milk, cheese, wine, and beer, all of which are dietary staples globally. These foods, mainly cereals, serve as primary sources of OTA contamination. Table 2 summarizes references concerning OTA occurrence from 2010.

Included in Table 2 are matrices, such as cereals and dehydrated products (e.g., raisins, dried figs, spices), that are directly subject to primary contamination in the field. By entering the food chain, farm animals convey OTA into meat, milk, and processed products (e.g., wine and beer), resulting in the intake of OTA by humans. Obviously, storage in the presence of moisture and permissive temperatures for the growth of OTA-producing microorganisms increases the initial contamination. In fact, environmental factors, such as humidity, temperature, and water activity, play critical roles in OTA formation [109]. Therefore, OTA presence has been documented across continents, including Asia, America, Africa, and Europe. In any case, beyond the well-founded perception of the occurrence of OTA in tropical and subtropical regions with warm humid climates, accurate statistical surveys of identical products demonstrating greater contamination in specific countries are not available, especially because warm, humid climate areas are present in many countries in temperate zones, and also because, for example, the OTA spoilage of cereals also occurs in Canada during winter wheat storage, with OTA levels up to 360 µg/kg in grain clumps close to manhole openings [81]. In addition, transport from major grain-producing countries to importing ones is usually by sea, where the high humidity or rewetting may contribute to the occurrence of OTA.

When considering the overall dietary exposure to OTA in Europe, wine ranks as the second most significant source after cereals [31]. Grapes damaged by ochratoxigenic fungal infection, facilitated by the high sugar content, create an optimal environment for OTA synthesis during winemaking. Geographic and climatic variables influence OTA levels in wine, with contamination often occurring in the vineyard. Europe, being a prominent wine producer and consumer, has established a maximum OTA limit of 2 µg/L in wine through the European Commission Regulation 1881/2006 and following Regulations [32]. The maximum level (ML) for OTA is 3 µg/kg in roasted coffee and 5 µg/kg in soluble coffee [32], with coffee being the fifth largest source of OTA exposure in Europe after cereals, wine, beer, and grape juice [31]. In sixth position is cocoa, a key ingredient of chocolate and other food products. The EC has set an ML for OTA in cocoa at 3 µg/kg, 5 µg/kg for unprocessed cereal grains, and between 2 and 4 µg/kg for products derived from cereals, depending on the ingredients. In comparison, in Brazil, the maximum limit for OTA in coffee, cocoa beans, cereals, and cereal products is 10 µg/kg [110]. Although a significant portion of cocoa production occurs in Western Africa, reports of OTA contamination in cocoa are relatively limited [111]. Despite their status as nutrient-dense and widely consumed foods, fruits, dried fruits (e.g., figs, raisins), and nuts (e.g., almonds, pine nuts, hazelnuts, chestnuts, walnuts) are susceptible to OTA contamination [93]. Spices, commonly used in culinary preparations, are also prone to OTA contamination due to their production and storage conditions, even though their consumption is low and not comparable with the previously mentioned food items. In Europe, the OTA ML for dried spices is set at 15 µg/kg, while the limit for dried *capsicum* spp. is 20 µg/kg [32]. Furthermore, cereals constitute a significant component of animal feed, potentially leading to OTA accumulation in livestock and later in humans through the food chain. In fact, OTA was detected in milk; therefore, in the EU, the ML for OTA in infant formulates is 0.50 µg/kg [32]. Table 3 shows a comparison of the OTA maximum limits set in different countries, which indicates that the European Union [32], along with Brazil [110], Turkey, and Vietnam, have set stringent OTA limits for various plant products or processed products [112]. In contrast, the FAO WHO Codex Alimentarius merely suggests a limit of 5.0 µg/kg for unprocessed cereal grains and 20 µg/kg for *Capsicum* dried fruits [113]. Other countries, such as China, have set limits for only a few products (dehydrated fruits, cereals, and coffee), while Singapore has established an OTA ML of 2.5 µg/kg any food, in the same way Japan and Switzerland (excepting for baby food, 0.50 µg/kg) determined a unique ML of 5.0 µg/kg, while India fixed an OTA ML only for wheat, barley, and rye (20.0 µg/kg), and Canada stated limits only for swine and poultry food (0.2–2.0 µg/kg). Instead, the US set MLs for aflatoxins, deoxynivalenol, patulin, and fumonisins, but not for OTA [112]. Values set by law are, of course, indicative of the level of contamination and possible harm to health in different countries.

However, the USA Food and Drug Administration (FDA) covers several essential elements of food safety, such as routine monitoring and sampling of a wide range of food items for the presence of mycotoxins, including OTA. In fact, Mitchell et al. [114] report sampling for the presence of OTA throughout the United States so that exposure can be calculated based on OTA concentrations in food products and consumption for different age groups of the population. Because OTA was not detectable in most of the samples analyzed, except for nuts, breakfast cereals, infant cereals, and cocoa, the (lifetime) margin of safety (MOS) for the USA population, within 95% of consumers of all possible products, was >1, indicating negligible risk. The authors concluded that, even in the absence of OTA regulations, the exposure to OTA in United States would not be associated with a significant risk of adverse effects [114]. Concerning Europe, the European Food Safety Authority (EFSA) Panel on Contaminants in the Food Chain estimated the margins of exposure (MOEs), and MOEs calculated for OTA neoplastic adverse effects, particularly those for high consumers and breastfed infants, were less than 10,000, indicating a possible health concern [31]. Thus, in observance of the European precautionary principle, the OTA MLs appear justified.

## 5. OTA Analytical Methods

Because of the intricate nature of the food composition and the usual minuscule presence of OTA in food, it is necessary to undergo some form of sample processing before testing food samples. Sample preparation is often a pivotal and demanding phase in the entire analytical procedure, and the approach can vary depending on the specific food matrix.

Sampling is crucial when analyzing OTA and other mycotoxins in food, as it significantly impacts the reliability of results and determines whether a food batch complies with safety standards. To ensure representativeness and accuracy, carefully designed sampling plans are essential [115,116].

Sample preparation for OTA analysis in food involves separating OTA from the food matrix, thereby enhancing detection sensitivity and specificity [117,118]. This process is influenced by the OTA chemical properties, food matrix nature, and chosen detection method [115]. OTA is typically extracted using organic solvents like methanol, acetonitrile, or acetone, sometimes with water or an acidic buffer to improve efficiency [115,119,120]. Other solvents tested were ethyl acetate and isopropyl alcohol [121]. An appropriate solvent must possess high OTA extraction capacity and not interfere (be neutral) with the subsequent steps of separation, identification, and quantification. Specifically, Prakasham et al. [121] showed in their work that acetonitrile is the solvent with the highest extraction capacity (greater than 90%), compared with the other four solvents mentioned above, on coffee, tea, and soil samples, while methanol was the least efficient solvent. Moreover, applying Fe_3_O_4_ nanoparticles for the analysis of the presence of OTA in sultana raisin samples, chloroform was selected over carbon tetrachloride, dichloromethane, and ethyl acetate [122]. The extraction of OTA from black pepper was better with an 80:20 methanol/water mixture [123], while methanol alone was more efficient than 70:30 methanol/water for OTA extraction from spiked rice samples [124]. Therefore, the solvent or mixture of choice will depend on the matrix type and subsequent cleaning of the extract and the final detection method. Cleaning up the extract is essential to enhance specificity and sensitivity, thereby improving accuracy and precision [119]. Methods for cleanup include liquid–liquid partitioning and, more recently, solid phase extraction (SPE) and immune-affinity columns (IAC). SPE involves specific partitioning of the analyte between a solid adsorbent and an organic solvent [121], while IAC uses antibodies to selectively bind mycotoxins [125]. A method called Quick, Easy, Cheap, Effective, Rugged, and Safe (QuEChERS) simplifies extraction and cleanup using acetonitrile, salts, and dispersive SPE, making it a fast and cost-effective option with minimal solvent usage [126,127]. Four absorbents (activated carbon, amorphous graphite, graphitized carbon black, and octadecylsilane) suitable for SPE columns have been evaluated by Prakasham et al. [121], who concluded that the best matrix for cleaning samples is activated carbon. However, immunoaffinity columns represent an effective and robust tool for mycotoxin cleanup, but the prediction is that soon, molecularly imprinted polymers will be competitive with immunoaffinity-based materials [128,129].

Various food products, such cereals, coffee, chocolate, wine, beer, and dried fruits, can be evaluated for the presence of OTA using different methods/techniques.

Thin-layer chromatography (TLC) is a rapid screening method for OTA, in which a thin layer of a stationary phase is used to separate OTA based on their properties. Teixeira et al. [130] efficiently used TLC with a charge-coupled detector (CCD) for Brazilian red wine samples.

High-performance thin-layer chromatography (HPTLC) was introduced as an advanced analytical technology derived from TLC, known for its accuracy and efficiency [131,132].

High-performance liquid chromatography (HPLC) is a technique that is widely employed with a C-18 chromatography column as a stationary phase and a solvent as a mobile phase. This process separates molecules, and detectors record their retention times. Prior to HPLC, immunoaffinity column purification is often employed to remove impurities, ensuring accurate mycotoxin analysis [133,134,135].

Liquid chromatography coupled mass spectroscopy (LC/MS/MS) is an extremely sensitive and accurate technology for mycotoxin analysis, including OTA. Several researchers discussed the use of LC/MS/MS in OTA detection in animal-derived foods and its ability to detect OTA in various food and feed samples [136,137].

Polymerase chain reaction (PCR) and quantitative real-time *PCR* (qPCR), which are techniques used to quantitatively measure DNA or RNA in a sample, permit us to indirectly detect the presence of OTA by monitoring the gene expression associated with mycotoxin production, particularly in fungal species like *Aspergillus* and *Penicillium* [138,139,140].

Enzyme-linked immunosorbent assay (ELISA) is a widely used method for OTA detection, and a new, highly sensitive OTA ELISA has been developed using monoclonal antibodies. Proficiency tests and reference samples validate its accuracy. A direct ELISA format showed promise for OTA quantification in medicinal herbs, exhibiting a low IC_50_ value and strong correlation with HPLC, as well as a toxin-free ELISA, employing an anti-idiotypic nanobody as a surrogate standard, offer high sensitivity and accuracy, which is confirmed by HPLC correlation [141,142].

Table 4 shows a list of techniques for OTA determination in different matrices and their detection limits.

Chemical and electrochemical biosensors, including immuno, electrochemical, and optical types, based on nanoparticles or not, are effective tools for OTA detection. In fact, antibodies, aptamers, imprinted polymers, peptides, and DNAzymes have been employed as selective recognition receptors for the electrochemical biosensing of mycotoxins, including OTA [160]. Nanoparticles are utilized in colorimetric, fluorometric, enzymatic, and electrochemical assays, contributing to the precision and sensitivity of mycotoxin detection [160,161]. Biosensors are easy to use, require a minimal volume of sample, and are characterized by fast response, a limit of detection (LOD) up to 0.31 fg/mL, and a linear range of 1–50 ng/mL [162], or an LOD of 0.18 ng/mL and a linear range of 0.78–200 ng/mL for OTA [163]. The biosensors, being conceptually designed to be portable, allow for the on-site detection of OTA, making them attractive for the industrial market. Their extensive use is expected in the future, but, at present, biosensors are not yet commercialized. 

Infrared spectroscopy uses infrared light to detect OTA without the need for sample preparation. It is fast and nondestructive but faces challenges with heterogeneous food matrices [164].

Fluorescence polarization (FP) measures the rate of rotation of a fluorophore and is used for detecting low-molecular weight materials like mycotoxins in a solution. It has limited sensitivity compared to HPLC [165].

Electronic nose (EN) mimics the human olfactory system and detects volatile compounds. It is still in development for OTA analysis and faces challenges with nonvolatile mycotoxins [166].

## 6. Strategies for OTA Control

Preventing the formation of OTA has emerged as a significant focal point to safeguard consumers against its detrimental impact on their well-being. The economic detriments stemming from OTA, in addition to its adverse consequences on public health, have spurred scientific endeavors to discover methods for averting the genesis of this toxin in agricultural settings. Equally, researchers are exploring techniques to detoxify OTA from food products. The effectiveness of these techniques varies depending on the chosen methodology, the timing of implementation, and notably, the specific fungal species [167,168].

The implementation of advanced agricultural technologies and good agricultural, manufacturing, and storage practices can mitigate OTA contamination [19]. To prevent fungal growth in plants, employing good agricultural practices like proper soil cultivation, crop rotation, efficient irrigation in drought conditions, accurate use of fungicides and fertilizers, timely harvesting, and adopting fungus-resistant crop varieties is vital [169]. Maintaining product quality during harvest necessitates using dry containers to manage agricultural produce and prevent fungal growth [170]. Inadequate preharvest methods can lead to fungal contamination and the buildup of OTA mycotoxin in stored agricultural products, particularly due to fungi like *P. verrucosum* and *A. flavus* infiltrating plants preharvest [171]. Postharvest techniques are essential for addressing OTA mycotoxin post-formation [172].

The control of postharvest OTA contamination revolves around two key strategies, as follows: firstly, the eradication of fungus-infected items, and secondly, the application of a spectrum of treatment methods to the products. These essential practices are incorporated into the storage and distribution phases of the food and feed supply chain. Additionally, this approach extends to the implementation of antagonistic microorganisms such as yeast, fungi, and bacteria, which serve as a shield against OTA production within consumables. Notably, the mere presence of fungus-infected items does not necessarily indicate the concurrent presence of OTA. Instead, the emergence of OTA hinges on a specific set of conditions encompassing nutrient availability, temperature, O_2_ levels, moisture, and time, all of which must coexist [173].

The methods used to decontaminate mycotoxins in food products should not only inactivate, destroy, or eliminate the toxin but also maintain the nutritional quality of the food, which should be acceptable for both human and animal consumption and should not significantly alter the technological properties of the product. It is important to note that there is no one-size-fits-all decontamination method that can be applied to all mycotoxins, as highlighted by Kamle et al. [19]. To address this challenge, decontamination strategies have been developed to specifically reduce OTA levels in food items. These strategies primarily rely on biological approaches that have no adverse effects on human health or the environment. This approach involves harnessing natural compounds like phenolic compounds and essential oils (Eos), which are derived from plants, as outlined in the studies by Dammak et al. [174] and El Khoury et al. [175]. Moreover, the use of *Bacillus amyloliquefaciens*, as demonstrated by Chang et al. [176], and lactic acid bacteria (*Lactobacillus plantarum*, *L. graminis* and *Pediococcus pentosaceus*), as shown in the research by Belkacem-Hanfi et al. [177] and Taheur et al. (*Lactobacillus kefiri*, *Kazachstania servazzii* and *Acetobacter syzygii*) [178], offers a safe and effective means of decontaminating food. Additionally, the application of bifunctional enzymes has proven to be a remarkable method for targeting specific mycotoxins, as discussed in the study by Azam et al. [179].

### 6.1. Chemical Methods

#### 6.1.1. Synthetic Fungicides

The initial phase in controlling fungal contamination involves the strategic application of fungicides in cultivated fields, a critical step that must ensure postharvest product quality, as stressed by Amiri et al. [180]. Throughout the years, fungicides containing chemical compounds like benzimidazoles, aromatic hydrocarbons, and sterol biosynthesis inhibitors have played a pivotal role in agricultural practices, effectively combating various plant diseases. Certain fungicides such as pyrimethanil and the cyprodinil/fludioxonil combination have demonstrated remarkable efficacy in hindering fungal growth and reducing the production of OTA. The latter combination, which is particularly highlighted in studies by Belli et al. [181] and Lagogianni and Tsitsigiannis [182], has shown promising results in Mediterranean countries. However, the indiscriminate and excessive application of fungicides has resulted in the emergence of pathogenic strains that are resistant to these treatments [183]. Consequently, repeated fungicide treatments have become necessary, leading to an increase in toxic residues in food, especially in the case of nonbiodegradable or poorly biodegradable fungicides. Considering these complex issues, the imperative to urgently develop novel, safe, and biodegradable alternatives that remain effective and are economically sustainable over time is evident. This need arises from the necessity to address the limitations and adverse consequences associated with conventional chemical treatments, thereby fostering the exploration of sustainable and safer approaches in the ongoing battle against fungal contamination in agriculture.

#### 6.1.2. Chemical Degradation of OTA

The strategy for detoxifying food commodities contaminated with OTA involves chemical methods to transform the toxin into other harmless substances. Common techniques include ammonization, alkaline hydrolysis, ozonation, and the use of compounds like bisulfites, acids, and oxidizing agents (Table 5).

Ammonization has historically been a widely utilized method for degrading OTA in various grains, such as corn, wheat, and barley. Despite its effectiveness in reducing OTA levels, this process significantly compromises the taste and quality of the treated foods. Browning of cereals and the loss of essential amino acids are among the noticeable drawbacks, as highlighted by Bhatnagar et al. [190]. Consequently, due to these adverse effects on both sensory perceptions and nutritional value, ammonization is no longer recommended as a suitable method for OTA detoxification. Alkaline hydrolysis, utilizing hydrogen peroxide (H_2_O_2_) and sodium hydroxide (NaOH), has shown potential in degrading OTA. However, its practical application is limited by the negative impact it exerts on the sensory and nutritional characteristics of the treated materials. Ozone treatment (O_3_), when applied briefly, has been an effective method in eliminating OTA in hazelnuts and various plants, as reviewed by Afsah-Hejri et al. [191]. Ozone, which is recognized for its potent oxidizing properties, effectively eliminates fungi and bacteria and aids in food preservation. However, ozone treatment can cause physicochemical changes in the treated food so that it is necessary, prior to industrial use, to test the effect on different matrices [191]. This is because it is necessary to control the oxidation (and peroxidation) of lipids because ozone is able to trigger a rapid accumulation of reactive oxygen species (ROS) in plant tissues [192], causing in turn, negative effects ranging from color changes and the loss of vitamin C in fruits and vegetables to changes in some quality parameters and the formation of an unpleasant smell when O_3_ is applied to flour [193]. Conversely, it is reported that ozone treatment limits lipid oxidation, inhibits the formation of volatile compounds [194], and enhances the production of antioxidant- and stress-related secondary metabolites (e.g., polyphenols) [195]. Finally, ozone usage is also restricted because it is a toxic gas that demands a controlled and enclosed environment for its application, with precise dosing to prevent potential harm to human health, as indicated by Akbar et al. [196].

### 6.2. Physical Methods

Research has revealed novel insights into physical detoxification methods, specifically addressing OTA contamination. Initially, sophisticated cleaning methods like microfluidization have undergone scrutiny to improve the extraction of contaminants from harvested grains. This refinement enhances the efficiency of critical processes, such as husking, peeling, and dust particle elimination. The investigation into microfluidization and analogous advanced cleaning techniques seeks to enhance the efficacy of the decontamination process. By employing high-pressure fluid dynamics, these methods ensure a more comprehensive removal of contaminants, thereby mitigating the risk of the presence of OTA in food products. This not only bolsters the safety of the food supply but also contributes to the overall quality and purity of the end products [197]. The optimization of existing processes through advanced cleaning techniques underscores the dedication of recent research to advancing food safety standards. In the face of evolving technology, the ongoing exploration of innovative methods becomes pivotal to guaranteeing the effectiveness of detoxification processes and, consequently, upholding public health (Table 6).

Regarding heat treatment, OTA displays resistance to heat and can withstand various food processing methods, such as roasting, blending, and cooking, up to a certain limit [203,204]. Based on earlier research findings, it has been observed that while OTA exhibits thermal stability at temperatures of 180 °C and higher, its activity tends to diminish [31]. Aguilar-Alvarez et al. [205] indicated that only 20% of OTA can be degraded through exposure to 100 °C for 160 min or 150 °C for 32 min. Consequently, this approach unavoidably exerts a substantial adverse effect on food quality and is exclusively applicable in the preparation of specific food items, such as coffee roasting. The reduction of OTA at high temperatures and the formation of its degradation products were investigated by Cramer et al. [206]. They demonstrated that when OTA was exposed to temperatures of 175 °C and above, its levels decreased primarily due to the formation of a diastereomer called 2′R-OTA. Sueck et al. [207] observed similar changes, with OTA isomerizing at 120 °C. Up until now, 2′R-OTA has been detected in food items that underwent high manufacturing temperatures. Although the levels of this diastereomer were low, it was confirmed to be present in products like coffee, cocoa, bread, and others [208,209,210]. The efficacy of eliminating OTA from food at elevated temperatures is suboptimal. This is primarily because high-temperature treatments lead to the pyrolysis of other active constituents, especially for conventional food processing temperatures in the range of 80 to 121 °C [200].

Several studies have investigated the use of cold plasma treatment for OTA decontamination. Ouf et al. [211] conducted an experiment in which they intentionally contaminated date palm fruits with *A. niger* and then subjected them to a 7.5-min treatment using a cold argon plasma under double atmospheric pressure conditions. This treatment led to the complete elimination of OTA. However, because argon (Ar) is a costly noble gas and the 7.5-min treatment duration is relatively lengthy, Durek et al. [212] observed that subjecting barley to CO_2_ plasma generated through a diffuse coplanar surface barrier discharge (DCSBD) resulted in an increase in OTA production from 49  ±  13.8 ng/g (in the control group) to 72.9  ±  45.8 ng/g after a 3 min treatment. Hoppanova et al. [213] found that OTA production showed a significant increase during the initial 4 days of incubation after treatment with plasma for 60 and 90 s. However, after seven days of incubation, the OTA production was lower than that in the control group. The final yield of OTA in plasma-treated samples was observed to be lower compared to untreated samples. It is important to consider various process conditions like gas composition and storage time in practical applications. Nonetheless, there are also recent studies indicating that plasma treatment can lead to a reduction in OTA content in food; Casas-Junco et al. [214] have employed helium plasma to treat OTA in coffee and noted that a 30 min plasma treatment resulted in a 50% reduction in OTA content and a shift in toxicity from toxic to slightly toxic; a similar OTA reduction (53%) was obtained by Guo et al. [215] for rice grains. Therefore, cold plasma remains a promising method for OTA decontamination because it is very effective against microbial activities [215].

In addition, gamma ray irradiation is effective in reducing OTA levels in contaminated corn by disrupting OTA aromatic ring double bonds through the generation of highly reactive radicals. Electron beam irradiation (EBI) possesses the ability to degrade OTA, and the degradation efficiency increases in an irradiation dose-dependent manner, as demonstrated by Peng et al. [216] in aqueous solution. Their findings indicated a degradation process that conforms to a first-order kinetic model. However, the drawbacks of this technology are that the degradation of OTA is influenced by several factors, including irradiation dose, composition of the food matrix, and toxin initial concentration. Additionally, this process may affect the nutritional content/value of food, reducing the content of vitamins, proteins, unsaturated fatty acids, and probiotics [217,218]. Regulatory considerations have evolved alongside these advancements so that a more nuanced approach to dosage is recommended. The permissible dose limit of 10 kGy is being reevaluated based on a comprehensive risk assessment framework, considering both OTA reduction and potential byproducts [219].

Pulsed light (PL) stands as an innovative nonthermal technique for sterilization. This method employs brief bursts of high-intensity, wide-spectrum light energy (λ ranging from 200 to 1100 nm) lasting from 0.1 to 1 s. Its purpose is to inactivate the microbial load in food and, more recently, for the degradation (removal) of toxic molecules. This approach offers benefits, such as sterilization, safe operation, and reduced environmental impact. The mechanism behind the efficacy of PL encompasses photochemical, photothermal, and photophysical effects [220,221,222]. Wang et al. [223] explored the degradation effect of OTA in grape juice by PL, and the highest degradation rate of 95.29% was achieved after optimizing the process using response surface methodology (RSM); the treatment transformed OTA into less toxic compounds like OTα and phenylalanine.

Ultraviolet radiation (UV) has also been used both for inhibiting the growth of ochratoxigenic fungi and detoxification of OTA. Sumbal et al. [200] showed that UV irradiation for 1 h on poultry feed reduced the OTA concentration from 500 μg/kg to 100 μg/kg and achieved a level close to zero after 8 h. More recently, Zhang et al. [224] showed that *A. carbonarius* and *A. ochraceus* fungal growth and OTA production were significantly reduced using short-wavelength blue and UV-B light, which both promote OTA degradation and reduce OTA biosynthesis.

Simple mechanical techniques like sorting, cleaning, and milling play a pivotal role in significantly reducing OTA levels, thereby augmenting the safety of the final food product. One crucial advantage lies in the ability of such physical methods to preserve the nutritional quality of the food, as they typically do not involve chemical alterations in the nutrient components. This ensures the overall quality of the food. Another commendable aspect is the environmentally friendly nature of these physical methods, which do not involve chemicals or additives. This aligns with sustainable practices, addressing both consumer concerns regarding food safety and environmental impact. Moreover, the versatility of physical methods allows their application to various food types, rendering them adaptable and effective across different commodities susceptible to OTA contamination.

Despite these advantages, it is imperative to acknowledge certain limitations associated with physical methods for OTA contamination. A significant constraint is an incomplete elimination of mycotoxins, leaving a residual contamination. Additionally, physical methods show a low ability to treat mycotoxins present inside the food matrix, primarily addressing surface contamination. The impact on product quality is another general issue, especially for methods like milling or grinding, which may alter the texture, appearance, or flavor of the final product. Striking a balance between mycotoxin reduction and maintaining product quality can be a complex challenge full of nuances. Furthermore, the high initial investment required for advanced physical methods, such as sorting technologies or specialized equipment, may pose a limiting factor, particularly for smaller producers. Lastly, the applicability of physical methods to certain foods may be reduced, especially when dealing with delicate fruits or vegetables whose quality can be easily compromised. Therefore, for the reduction of OTA through physical methods, a very careful choice is needed considering the specific characteristics of the food product and production scale.

### 6.3. Adsorption by Physical–Chemical Agents

A different approach to removing OTA from agricultural products involves using adsorbent materials. These materials can bind OTAs, rendering them immobile. This process aims to eliminate OTA through a subsequent simple filtration. Adsorbent materials can be grouped based on their source (Table 7), including (i) natural materials such as jujube stones, oyster mushroom powder, dried fruit shells, olive pomace, and other fruit wastes [225,226,227]; (ii) inorganic minerals mainly represented by activated carbon, aluminum silicate, hydrated sodium calcium aluminosilicate, bentonite, zeolite, diatomaceous earth, and sea foam [228,229,230]; and (iii) organic synthetic materials such as modified silica gel, cellulose polymers, potassium caseinate, and cross-linked chitosan [231,232].

Among the various types of adsorbents studied, activated carbon and potassium caseinate have demonstrated the highest efficacy. When activated carbon is employed, around 90% of OTA is removed from red wine, while using 150 g/L of potassium caseinate results in an 82% reduction [238]. However, activated carbon should be used with caution because it also absorbs anthocyanins and other polyphenols from the wine. Similarly, the effectiveness of using oak wood fragments for treatment varies in relation to the quantity of wood chips or powder utilized [239]. Research by Olivares-Marín et al. [240] suggested that activated carbon derived from cherry stones could potentially eliminate up to 50% of OTA in wine without negatively impacting the overall polyphenolic index and color intensity. Conversely, other adsorbents like bentonite, cellulose acetate esters, polyvinylpyrrolidone, cholestyramine, and polygel have proven to be poorly effective in removing OTA [228,234,241,242,243], but *Lactobacillus plantarum* encapsulated in a polymeric matrix composed of polyvinyl alcohol and alginate, at a concentration of 0.5 g/mL, was able to remove over 50% of the OTA without substantially affecting the presence of total phenols in red wine [244]. Adsorption using physical–chemical agents has emerged as a promising strategy for the control of OTA contamination, demonstrating notable advantages in mycotoxin management. A key strength lies in the versatility of physical–chemical agents, allowing their application across a broad spectrum of food products and feed materials. This methodology preserves the nutritional quality of treated products; nevertheless, it could cause alterations in taste, color, or texture. However, selectivity in adsorption poses a potential limitation, restricting efficacy against a broad spectrum of mycotoxins and necessitating a nuanced approach for comprehensive mycotoxin control. The regeneration and reuse of adsorption agents presents drawbacks, as the saturation of adsorption sites over time may diminish the efficacy. Economic factors, particularly the cost associated with some agents, influence the feasibility of adoption, especially for small-scale producers, while environmental considerations, such as the origin and disposal of adsorbent substances, require careful evaluation to ensure sustainable practices in mycotoxin mitigation strategies.

### 6.4. Biocontrol Strategies for OTA Control

The negative drawbacks of using physical and chemical methods on the overall quality of food products has raised concerns within the field of toxicology. In response, the use of biological techniques that pose no threat to human health or the environment has been suggested [245]. They involve the utilization of the capabilities of microorganisms like bacteria, yeasts, and fungi, as well as natural compounds derived from plants, such as phenolic compounds and essential oils, to degrade, alter, or absorb OTA or limit the biosynthesis process. This represents a significant shift away from conventional methods, signifying a move towards safer, more environmentally conscious practices aimed at preserving food safety and quality.

#### 6.4.1. Microorganisms and Enzymes

The exploitation of the ability of microorganisms to naturally resist OTA contamination has gained more attention in recent years. Table 8 shows the main results obtained in the attempt to solve OTA contamination.

The basic mechanism of OTA degradation involves the enzymatic breakage of its amide bond catalyzed by carboxypeptidase, thereby generating significantly fewer toxic intermediary products. Carboxypeptidase acts specifically on the amide bond within the OTA molecule, resulting in byproducts like Otα and L-β-phenylalanine, which are considered less hazardous than the original compound. In fact, by altering the molecular structure of OTA, carboxypeptidase degrades it into smaller, less harmful components, playing a crucial role in this degradation process. As highlighted in the study by Wei et al. [269], this enzymatic action is a pivotal step in OTA’s natural degradation process, reducing its toxicity. This knowledge is crucial for developing effective strategies to combat OTA contamination across different settings. Actinobacteria strains are characterized by a remarkable capacity to address OTA contamination, making them able to completely degrade OTA in liquid media and reduce OTA levels in solid media. However, it is important to recognize that degradation methods can vary, leading to differences in the observed breakdown products. Detecting common byproducts of degradation might not always be possible, indicating a few mechanisms of action. An example of one such mechanism involves the facilitation of OTA degradation through the hydrolysis of the OTA lactone ring, conducted by microbial enzymes like lactonohydrolases or esterases, as discussed in Campos-Avelar et al. [274]. This process is pivotal in dismantling OTA structure. The varied nature of microbial OTA degradation pathways indicates a microbial adaptability that is crucial in addressing OTA contamination in diverse environments. Understanding these multifaceted mechanisms is critical in crafting comprehensive and effective strategies to combat OTA contamination in various scenarios, covering agricultural fields to food processing and storage facilities, thereby ensuring safer and healthier conditions for consumers. Lactic acid bacteria (LAB) are renowned for their probiotic properties and various beneficial effects. Muhialdin et al. [275] suggested that specific strains of these bacteria have the potential to effectively bind or adsorb OTA. When these bacteria adsorb OTA, they essentially trap or bind the toxin, preventing its absorption by the body upon ingestion. This action could potentially mitigate the adverse effects of OTA on human health. The efficacy of this adsorption process holds significant promise for enhancing food safety. If certain strains of lactic acid bacteria can be harnessed to minimize OTA contamination in food products, it might provide a natural and potentially safer method to control the risks associated with this mycotoxin. In a study conducted by Fuchs et al. [276], *Lactobacillus acidophilus* VM20, a specific strain of *Lactobacillus* bacteria, demonstrated an exceptional ability to convert a substantial portion of OTA within a remarkably brief timeframe. The research reported an impressive conversion rate of 96% of OTA, even at a high concentration of 1 μg/mL, achieved in just 4 h, which is a very short time for toxin detoxification. Similar data were obtained by Luz et al. [254] who registered OTA reduction values of up to 97% and 95% with two different strains, *L. rhamnosus* CECT 278T and *L. plantarum* CECT 749; OTA reduction was both enzymatic and via adsorption on the cell wall. Similar results were obtained by Domínguez-Gutiérrez [277] when testing the effect of *Lactobacillus plantarum* cultures on the inhibition of the *A. carbonarius* growth and OTA production. However, the effectiveness could vary based on the specific bacterial strain, OTA concentration, and the type of food product. Further research and studies are required to better comprehend the mechanisms of OTA adsorption by these bacteria and to verify the safety and efficacy of this approach before it can be implemented as a solution. The study conducted by Shi et al. [278] presented fascinating findings regarding the ability of *Bacillus subtilis* CW14 in degrading OTA. They reported that the cell-free supernatant of this bacterium was remarkably effective, degrading an impressive 97.6% of OTA at a concentration of 6 μg/mL within a 24 h period. Notably, no byproducts of the degradation process were detected. Additionally, the research highlighted that both live and autoclaved *B. subtilis* CW14 cells bound more than 60% of OTA. This research presents intriguing possibilities for mycotoxin control in food safety. The effectiveness of the *B. subtilis* CW 14 cell-free supernatant in degrading OTA without generating byproducts is a significant finding. The understanding of the mechanisms involved in degradation and adsorption is vital for considering the application of such bacterial strains, which could potentially serve as a natural and safer strategy to minimize the risks associated with mycotoxin contamination. The investigation led by Rodriguez et al. [279] unveiled the capacity of *Brevibacterium casei* RM101 to thoroughly decompose OTA. Specifically, this bacterium accomplished an outstanding 100% degradation of OTA at a concentration of 40 μg/mL within 10 days. This discovery holds significance as it highlights the robust capability of *B. casei* RM101 to completely degrade OTA, suggesting its potential as an effective solution for managing and eradicating this mycotoxin from diverse food products. Upadhaya et al. [280] highlighted how *Eubacterium biforme* MM11 also effectively eliminates OTA. Within a 12 h incubation period, *E. biforme* MM11 successfully removed 77.1% of OTA at 0.1 μg/mL. This outcome underscores the potential of this bacterial strain as a means of bioremediation to minimize OTA contamination. Wei et al. [281] investigated how N-acetyl-L-cysteine (NAC) affects OTA and the combined impact with *Cryptococcus podzolicus* Y3 in degrading OTA. N-acetyl-L-cysteine, which is known for its antioxidant properties, has been under investigation for its potential to mitigate the toxic effects of OTA. The study findings unveiled that exposing *C. podzolicus* Y3 to NAC 10 mM resulted in significant improvements of the rate of degradation into Otα. Specifically, the rates of OTA degradation improved by 100% after 24 h and by 92.6% after 48 h. This highlights the substantial enhancement in *C. podzolicus* Y3′s capacity to break down OTA in the presence of NAC. Zeidan et al. [282] emphasized *Burkholderia cepacia*’s ability to combat various mycotoxigenic fungi. This bacterium has shown effectiveness against a broad spectrum of fungal genera and species known for producing mycotoxins. Additionally, the research noted that the liquid part/supernatant of *B. cepacia* culture had the capacity to hinder the production of OTA in *A. carbonarius* [282]. The antagonistic properties of this bacterium against mycotoxigenic fungi and its capability to limit mycotoxin creation represent another significant discovery in the context of food safety. Some of the strains known for their OTA-degrading abilities also displayed antagonistic activity. Specifically, *B. subtilis* CW14 [283] and *B. megaterium* [284] not only exhibited OTA degradation but also effectively inhibited fungal growth.

In addition to bacteria, fungi serve as crucial resources for degrading OTA. In a study conducted by Varga et al. [285], it was found that the atoxigenic *A. niger* CBS 120.49 strain could efficiently convert OTA (2.5 μg/mL) into Otα within just 5 days when cultivated on solid medium. This process was quicker compared to the 7 days needed when using a liquid medium. During a 7-day growth period on solid substrates, OTα was further converted into an unidentified substance. Among 55 strains of *Rhizopus* and *Mucor*, several strains of *Rhizopus* were able to break down OTA (7.5 μg/mL) to levels that could not be detected within 10 days when grown in a liquid medium. The *A. niger* (CBS 120.49) strain was especially effective, breaking down more than 90% of OTA within 6 days. In contrast, the *R. stolonifer* var. *stolonifer* strain TJM 8A8 required 12 days to achieve similar degradation in a liquid medium. In a separate trial, only *R. stolonifer* var. *stolonifer* TJM 8A8 managed to break down 96.5% of OTA (7.5 μg/g) within 10 days when applied to dampened wheat [286]. In addition, several strains of *A. niger*, *A. carbonarius*, *A. japonicas*, *A. wentii*, *A. clavatus*, *A. ochraceus*, *A. pullulans*, *A. versicolor*, *Alternaria*, *Botrytis*, *Cladosporium*, and *Penicillium* have demonstrated varying capacities for OTA degradation, leading to the formation of OTα and phenylalanine byproducts [287,288]. Various yeast strains and yeast-derived products are under scrutiny to determine their efficacy in actively countering or reducing OTA levels across diverse food products. Understanding how yeast can effectively inhibit or diminish OTA in food items represents a critical advancement in enhancing food safety measures. 

Ponsone et al. [289] reported the substantial capability of *Kluyveromyces thermotolerans*-RCKT4 and *K. thermotolerans*-RCKT5 in reducing the growth of *A. niger* and *A. carbonarius* and OTA accumulation in grapes, from as low as 3% up to a remarkable 100% reduction. These results encourage further examination and potential consideration of yeast strains for use in food processing and agricultural practices against mycotoxins. Thereafter, Ponsone et al. [290] evaluated two *Lanchancea thermotolerans* strains’ efficacy in degrading OTA accumulation by ochratoxigenic fungi over three years in both greenhouse and field settings. Their research demonstrated that these yeast strains effectively controlled OTA accumulation in wine grapes at harvest by 27% to 100%. *Saccharomyces cerevisiae* have proven effective in diminishing OTA levels in musts and wines by a range of 20.3% to 76.4%. Other yeast species, such as *Cyberlindnera jadinii*, *Candida friedrichii*, *Candida intermedia*, and *Lachancea thermotolerans*, also contribute to lowering OTA levels, achieving reductions of 20% to 67.5%. Additionally, *Trichosporon mycotoxinivorans* exhibits complete degradation of OTA during the fermentation process [23,291]. *Kloeckera apiculata* strains 3187, 3188, 3189, 3197, 3198, and 3200 can eliminate between 25 and 40% of OTA (0.006 μg/mL) over 20 days [292]. In addition, Zou et al. [151] demonstrated that an employed UV–mutated *Aspergillus niger* (FS-UV-21) exhibited an impressive OTA degradation efficiency up to 89.4% under specific conditions, resulting in a substantial reduction of thein OTA cytotoxic effects of treated food. Finally, Fiori et al. [293], tested the capacity of viable and nonviable yeast strains to adsorb OTA, demonstrating that strains of *Candida friedrichii*, *C. intermedia*, *Lachancea thermotolerans*, and *Cyberlindnera jadinii* within an 8-day period exhibited OTA adsorption percentages of 70, 73, 75%, and no significant adsorption, respectively, whether vital, and 72, 74, 84, and 82% after autoclaving, respectively. Notably, a significant disparity in OTA adsorption capacity between the viable and nonviable yeast strains *Cyberlindnera jadinii* was observed in this research.

**Figure 3:**
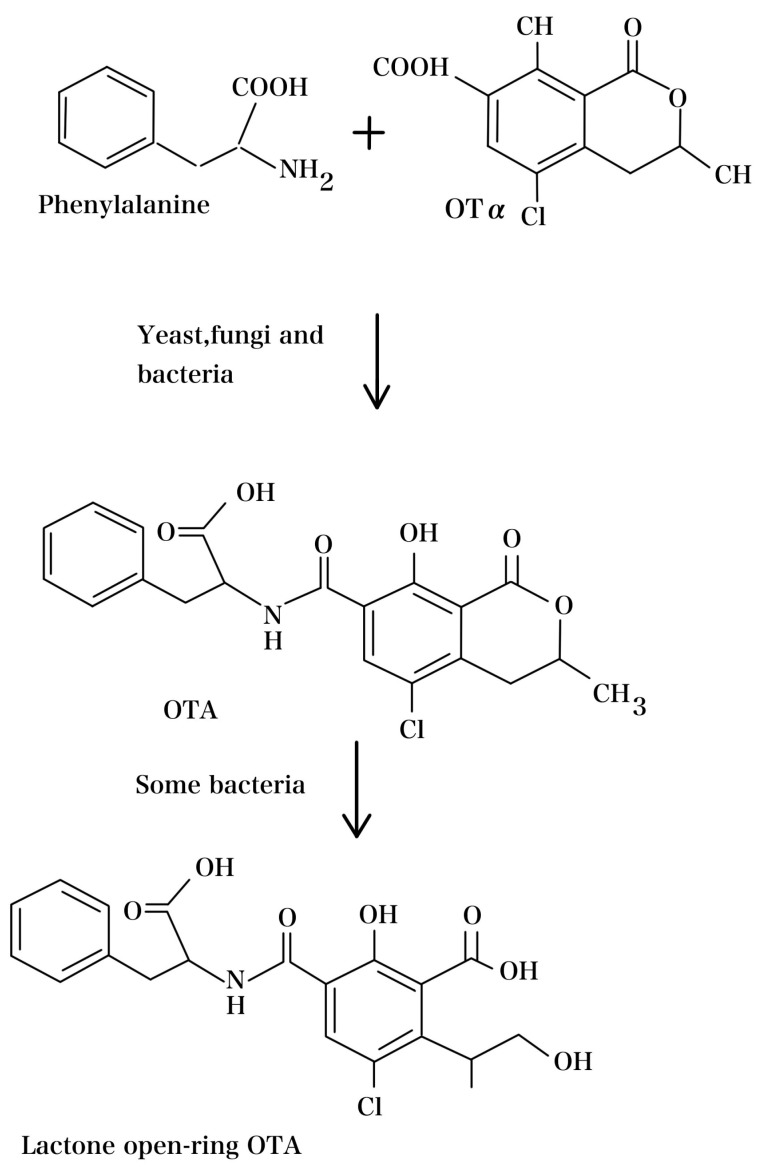
The biodegradation mechanism of OTA (redrawn after [296]).

The primary mechanism for OTA degradation relies on enzymatic activity. It is worth noting that these enzymes can exist in either extracellular or intracellular forms [294]. The degradation process involves a range of enzymes, including lipases, laccase, amidases, dioxygenases, ochratoxinases, and proteases, all of which can catalyze the breakdown of OTA. Additionally, researchers have developed a recombinant enzyme with zearalenone hydrolase and carboxypeptidase capabilities for OTA degradation [271,295]. These enzymes have the capacity to degrade the OTA–amide bond or open the lactone ring, leading to the production of less toxic compounds, such as OTα (Figure 3).

Dobritzsch et al. [297] showed that a purified recombinant ochratoxinase was more efficient in hydrolyzing OTA than carboxypeptidase A (CPA) and Y (CPY), the two previously known enzymes capable of degrading OTA; the recombinant enzyme is thermostable and has optimal activity at pH 6 and 66 °C. Commercial enzymes protease A and pancreatin achieved 87.3% and 43.4% degradation, respectively, on OTA 1 µg/mL, while commercial prolyve PAC managed only 3% degradation at 37 °C and pH 3 [298]. Moreover, the purified metalloenzyme from *A. niger* possesses an OTA hydrolytic activity 12.8 times higher than CPA at 37 °C and pH 7.5, showing 36 U/mg activity [299]. In another study conducted by Abrunhosa et al. [300], commercial carboxypeptidase Y from *S. cerevisiae* exhibited its maximum hydrolytic activity on OTA (1.1 µg/mL) at pH 7.5 and 37 °C; however, the enzyme displayed relatively low specific activity at pH 5.6, as only 52% of OTA was transformed into OTα following a five-day incubation period. Cho et al. [288] found that crude enzymes from *A. tubingensis* strain M036 removed, within 24 h at 25 °C, 97.5% and 80.3% of the OTA present (0.04 μg/mL) at pH 5 and 7, respectively. Stander et al. [301] investigated the hydrolytic activity of a lipase preparation from *A. niger* on OTA, observing that the enzyme was able to convert OTA into OTα and phenylalanine; the purified enzyme achieved complete hydrolysis of 80 µg/mL of OTA in a 120 min incubation period at 37 °C pH 7.5. Dalsgaard et al. [302] patented an amidase and a related feed/food additive capable of degrading OTA. This enzyme, termed amidase 2, encoded by *A. niger*, effectively reduced the OTA concentration by 83% in a 300 µL reaction mixture containing 160 ng/mL amidase 2 and 50 µg/mL OTA. Furthermore, when tested in contaminated milk, the patented enzyme reduced the OTA content from 47 ppb to <2 ppb in just 2.5 h. Similarly, in corn flour, it lowered the OTA concentration from 38 ppm to <2 ppb after 20 h of incubation. Peng et al. [303] isolated from *Brevundimonas naejangsanensis* (strain ML17) four novel OTA and OTB degradases, denoted as BnOTase1, BnOTase2, BnOTase3, and BnOTase4. These enzymes facilitate the degradation of OTA and OTB into OTα and OTβ by enzymatically hydrolyzing the lactone molecular structure, achieving degradation rate of up to 100%; the K_m_ of the recombinant enzyme that was more active on OTA was 19.38. Ma et al. [304] adopted immobilization technology to enhance the stability and degradability of CPA. By embedding CPA within zeolitic imidazolate framework materials, they achieved significant improvements. The immobilized enzyme demonstrated the ability to be reused over 10 times, and it exhibited a 30.7% higher degradation rate of OTA compared to free CPA. Finally, Luo et al. [273] reported that a recombinant ADH3 from *Stenotrophomonas acidaminiphila* (at 1.2 µ/mL) completely degrades 50 µ/L OTA within 90 s; the catalytic efficiency of the enzymes was estimated as 35,000 times higher than those of commercial carboxypeptidase A (CPA).

However, the direct employment of microorganisms capable of carrying out enzymatic reactions for OTA degradation on agricultural products and processed foods presents numerous advantages. These organisms offer a biologically sustainable approach to combatting OTA and are capable of either degrading the toxin or inhibiting the growth of its producing fungi, thus representing a natural alternative to chemical fungicides. Additionally, this method proves cost-effective and targets both OTA and OTA-producing fungi specifically, thereby minimizing harm to nontarget organisms and reducing ecological damage. Moreover, employing microorganisms for OTA control also has limitations. Effectiveness can fluctuate based on environmental factors like temperature, pH, and moisture. Regulatory barriers to approval and complex application methods are challenging, as is the search for a single microorganism that is effective against all OTA-producing fungi. Moreover, introducing new microorganisms into ecosystems could upset the existing microbial balance. Instead, enzymes represent a valuable tool in controlling OTA contamination due to their specificity in targeting OTA molecular structure and breaking it down into less harmful metabolites. Enzymes are safe and environmentally friendly because they are unable to reproduce and show versatility in degrading OTA; however, their activity also depends on temperature, pH, and moisture, and regulatory hurdles and cost concerns persist. Overall, the advantages of these biological control methods for managing OTA contamination in food are evident, but their limitations must be carefully taken into account for effective and safe implementation. Balancing these factors is crucial for advancing research, potential industrial integration, and securing regulatory approval in the domain of mycotoxin management.

In fact, a new emerging technique for the biological control of mycotoxins is the utilization of bacterial biofilms. In a recent study by Nahle et al. [305], the effectiveness of bacterial biofilms derived from *L. rhamnosus* was investigated for the removal of OTA from red grape juice. The assessment of OTA reduction in this beverage was conducted using ELISA, accounting for various influencing factors. The research unveiled the promising potential of this method for mycotoxin elimination. The application of bacterial biofilms, particularly those obtained from *L. rhamnosus*, offers an innovative and potentially impactful strategy to combat mycotoxin contamination, specifically in beverages like red grape juice. The study cited above contributes to the expanding realm of research exploring inventive methods for mycotoxin mitigation [305,306]. Not only does it demonstrate the efficacy of bacterial biofilms in reducing OTA and other mycotoxin levels but it also underlines the potential for these biofilms to be utilized in the food and beverage industry to uphold product safety and quality. Employing bacterial biofilms for mycotoxin removal signifies a new frontier in food safety strategies encouraging further investigations to refine this technique and potentially apply it to a broader array of food and beverage products.

#### 6.4.2. Plant-Derived Extracts for OTA Biocontrol

Significant scientific research has been devoted to exploring the potential therapeutic applications of plants and medicinal herbs, which are strategically utilized to address minor ailments. The overarching goal of these studies is to enhance our understanding of the intricate mechanisms through which the diverse array of active compounds, including essential oils and extracts, found in these botanical sources effectively counteract the presence of mycotoxins.

The mode of action of plant-derived extracts involves three main aspects that contribute to their inhibitory function. Firstly, the presence of hydroxyl (OH) groups capable of forming hydrogen bonds affects enzymes and intracellular functions. Secondly, these extracts interact with membrane enzymes, altering the rigidity and integrity of hyphal cell walls, thus impacting mold morphology. Thirdly, changes in cell membrane permeability, cytoplasmic granulation, and the rupture of cytoplasmic membranes play a role. These aspects are interconnected, with hydrophobic compounds crossing cell membranes to interact with cellular components, affecting both membrane and intracellular enzymes. Hydrophobic compounds can change membrane permeability for ions, leading to proton flow alteration, cell pH modification, and subsequent cellular disruption. The solubility of these compounds in the lipid phase of cytoplasmic membranes is crucial for their activity, thereby impacting microbial cell permeability.

Within the spectrum of these active constituents, phenolic compounds demonstrate a potent capability not only to slow down fungal growth but also to neutralize and inhibit the toxic effects associated with mycotoxin exposure [35]. Phenolic compounds can affect membrane proteins, causing structural deformations and functional changes [307]. Other effects include the disruption of proton motive force, interference with energy generation, enzyme inhibition, and substrate utilization prevention. These actions collectively lead to the inhibition of spore germination, mycelial growth suppression, and germ tube elongation. Plant compounds may influence the switch from vegetative to reproductive development through signal perception/transduction [308,309].

Passone et al. [310] analyzed the impact of plant essential oils (EOs) on OTA production by fungi belonging to the *Aspergillus* section *Nigri*. The primary aim was to discern the influence of different EOs on OTA accumulation and how environmental factors, particularly water activity (aw), contributed to these interactions. Notably, boldo (*Pëumus boldus*) EO exhibited inhibitory effects on OTA accumulation at a concentration of 2000 μL/L, suggesting its potential to effectively impede OTA production by ochratoxigenic fungi. However, the study uncovered intriguing complexities in the effects of poleo (*Lippia turbinata*) EO at different aw levels; at the high aw of 0.98, 1000 μL/L of poleo EO unexpectedly stimulated OTA production of the majority of *A. niger* and *A. carbonarius* strains. Moreover, clove (*Syzygium aromaticum*) EO demonstrated a pronounced inhibitory effect at a lower aw of 0.93, and at a concentration of 5000 μL/L, it achieved complete suppression of OTA production, suggesting its potential efficacy in inhibiting OTA synthesis, especially under conditions of lower a_w_. These results underscore the intricate relationship between EOs, environmental factors like a_w_, and OTA production by ochratoxigenic fungi. These findings hold promising implications for devising strategies aimed at controlling mycotoxin contamination in food products. In a 2019 study by Dammak et al. [174], the efficacy of EOs obtained from *Salvia officinalis*, *Lavandula dentata*, and *Laurus nobilis* (primary component 1,8-cineole), was tested for their potential to inhibit fungal growth and the production of OTA. *L. nobilis* EO demonstrated the highest inhibition of fungal growth, surpassing *L. dentata* and *S. officinalis*, with inhibition percentages of 47.82%, 37.92%, and 31.71%, respectively. Notably, *L. nobilis* EO exhibited a remarkable 88.87% reduction in OTA production. Moreover, in contact assays, both *L. nobilis* and *L. dentata* EOs displayed heightened antifungal and anti-ochratoxigenic activities compared to *S. officinalis*. Their minimum inhibitory concentrations (MICs) were approximately 0.3%, while *S. officinalis* EO exhibited a slightly higher MIC at 0.5%. Importantly, the MIC of 1,8-cineole, a key component in these EOs, was about twice that of *L. nobilis* and *L. dentata* EOs. These results underlined the varying effectiveness of different EOs and their components in inhibiting both fungal growth and the production of OTA. Particularly, *L. nobilis* EO emerged as notably effective in suppressing fungal growth and reducing OTA production. The data also highlight the potential utility of *L. dentata* EO against ochratoxigenic fungi.

Among EOs, starfruit seed EO has shown promise in significantly reducing OTA production. Researchers have noted a gradual reduction in OTA production with increasing doses of starfruit seed EO. Notably, this reduction did not solely hinge on the inhibition of *A. ochraceus* fungus growth, a fact supported by studies like the one conducted by Murthy et al. [311].

Scientific investigations have additionally spotlighted EOs as potential regulators of the genes involved in the synthesis of OTA. In the concentration range of 75–150 mg/mL, lemon EO has demonstrated a distinct capacity to impede OTA production in *A. ochraceus*; its disruption in OTA synthesis appeared to stem from the suppression of both OTA biosynthesis and its associated regulatory genes Hua et al. [312]. What is particularly intriguing is the mechanism of action, which does not merely hinder fungal growth but appears to directly interfere with the molecular processes responsible for OTA biosynthesis, highlighting the potential to target specific step of mycotoxin biosynthesis. Similarly, an independent study conducted by Ferrara et al. [313] revealed a noteworthy reduction in the expression of the polyketide synthase *AcOTApks* gene in *A. carbonarius* when exposed to a concentration of 0.065 mg/mL of two hydrocinnamic acids, ferulic acid and p-coumaric acid. These findings served as compelling evidence of the influence of specific compounds present in essential oils on the genetic expression of pivotal genes involved in mycotoxin biosynthesis. The observed marked reduction in the *AcOTApks* expression emphasizes the potential of EO compounds to disrupt the genetic pathways responsible for mycotoxin production. El Khoury et al. [314] performed a comprehensive investigation into the inhibitory impact of various EOs like fennel, cardamom, anise, chamomile, celery, cinnamon, thyme, taramira, oregano, and rosemary on the growth of *A. carbonarius* strain S402. Their findings unveiled a direct link between the reduction in OTA production and the suppression of two known biosynthetic genes involved in the OTA biosynthesis (*acOTApks* and *acOTAnrps*), as well as the *acpks* gene and the two regulatory genes (*veA* and *laeA*). In the presence of fennel EO at a concentration of 5 μL/mL, *acOTApks* experienced an impressive reduction of 99%. Moreover, the effects of these EOs on the transcriptional factors *veA* and *LaeA* were notable, showing significant decreases at the same 5 μL/mL concentration. Notably, the most substantial reductions in the expression of the *veA* and *laeA* genes were observed after the addition of EOs from fennel (30% and 40%), cardamom (71% and 70%), chamomile (94% and 96%), rosemary (95% and 91%), anise (87% and 80%), and celery (88% and 80%), respectively. Lately, El Khoury et al. [175] repeated the analysis of the effects of EOs and phenolic compounds on *A. carbonarius* S402. They observed that while specific compounds had a limited impact on the dry mass of the fungus, they significantly influenced OTA production. EOs derived from bay leaves, mint, and melissa notably reduced OTA production by 72%, 70%, and 80%, respectively. In contrast, phenolic extracts exhibited effectiveness in inhibiting OTA production but generally to a lesser extent compared to EOs, with fenugreek being a notable exception. In the case of fenugreek, its EOs at a concentration of 1 μL/mL increased OTA production by 45.1%, while its phenolic compounds inhibited it by 32.0%, but the toxin production was reduced by 60% with EO 5 μL/mL. In El-Desouky research [315], aqueous extracts obtained from *Sonchus oleraceus*, *Cichorium pumilum*, and *Portulaca oleracea* hindered the production of OTA by *A. ochraceus*. The inhibition determined by plant extracts (5 mg/mL) were notably high, 77.5%, 72.3%, and 85.2%, respectively. These results indicated the potential of the aqueous extracts, as well. Recently, Naz et al. [316] assessed the antifungal properties of eight eOs against *A. parasiticus* using cereal grains as substrate. The researchers emphasized the exceptional efficacy of *Cinnamomum verum* EO, which displayed the lowest MIC (1.04 μg/mL) among the EOs tested. EOs from *Syzygium aromaticum*, *C. verum*, and *Eucalyptus globulus* exhibited substantial antifungal effects, eradicating *A. parasiticus* spores within 30, 60, and 90 min, respectively, even in contaminated grains. Additionally, maintaining a 10% moisture content in broken rice resulted in minimal OTA presence (8.16 ng/g) in stored grains. In addition, the study noted an increase in OTA production with moisture levels up to 40%, but this trend reversed at higher moisture levels of 50% to 70%, but no increase in OTA was observed in grains treated with *C. verum* EO.

Nanoencapsulation formulations stand as a dynamic and evolving research field, showcasing a promising synergy between encapsulated substances and phytochemicals, thereby minimizing potential side effects. Opting for natural formulation-based antifungal agents presents a safer, more efficient, and eco-friendly approach in combating fungi and mycotoxins in agricultural settings, compared to conventional methods. This line of thought finds support from the research by El-Desouky and Ammar [317], Pushpalatha et al. [318], Dhakar et al. [319], Redondo-Blanco et al. [320], Sajid et al. [321], and Thipe et al. [322].

A significant area of interest in this domain involves the development of nanosponges derived from β-cyclodextrin, housing natural bioactive substances. These meticulously engineered nanosponges are crafted to impede the growth of harmful fungi and eradicate mycotoxins present in both food and feed. They are poised to serve as potential biofungicides, aiming to supplant synthetic pesticides. Studies conducted by Pawar et al. [323], Sherje et al. [324], Bhowmik et al. [325], Haimhoffer et al. [326], and Ananya et al. [327] further underscore the potential of these nanosponges in addressing mycotoxin-related threats. The combinatory potential of nanosponges with botanical or other compounds presents a promising avenue in tackling phytotoxic issues. For instance, encapsulated herbicides have exhibited success in combating parasitic weeds while minimizing adverse environmental impacts, as illustrated in the work of Asghari et al. [328]. The study by Appell and Jackson [236] investigated the efficacy of a polymer (nanosponge) composed of β-cyclodextrin and polyurethane in reducing levels of OTA in water and red wine. The lowering of OTA levels in red wine from 10 μg/L to the recommended limit of 2 μg/L is particularly noteworthy, as this is crucial for compliance with regulatory standards and ensuring the safety of the product for consumption. The study revealed the nanosponge maximum binding capacity of 220 μg OTA per gram of polymer. The higher binding capacity suggests that this nanosponge has a considerable affinity for OTA molecules. The use of β-cyclodextrin in the nanosponge is strategic due to its ability to form inclusion complexes with various molecules, which aids in the adsorption and removal of specific compounds like OTA. The combination with polyurethane might provide structural stability and enhance the overall effectiveness of the nanosponge. Appell et al. [329] presented an innovative methodology by creating a solid phase extraction sorbent utilizing a nanosponge formed from a mix of β-cyclodextrin and methylene bis-diphenyl diisocyanate in a 1:5 ratio. The primary aim was to evaluate the efficiency of this polymer in extracting OTA from wine and grape juice samples. Recovery rates within the range of 69.1% to 89.4% for OTA concentrations ranging from 0.5 to 20 ng/mL demonstrated the polymer effectiveness in extracting the toxin, spanning concentrations commonly encountered in these beverages. Additionally, the inclusion of methylene bis-diphenyl diisocyanate in the polymer likely contributes to the nanosponge structural stability and binding properties.

The application of plant extracts and EOs for controlling OTA contamination encounters several significant challenges as variability in their efficacy depends on the wide range of plant types, extraction methods, and the multitude of fungal strains involved in OTA production. Moreover, precise regulations regarding acceptable limits and the food use of essential oils are absent. Also, cost, availability, shelf life, and environmental impact are significant barriers to their practical application. The same is true for nanoencapsulation-coated plant extracts; the complexity of the encapsulation process, associated costs, and the need for continued research and development present significant obstacles for their implementation within food safety strategies.

## 7. Prospects and Application

A wide range of approaches, both scientific and technological, as well as regulatory and educational, need to be applied in the future to counter OTA contamination. This will take interdisciplinary cooperation, innovation, and a dedication to food safety to pursue the objective. Therefore, it will be necessary to take the following actions:

(a) Continuous improvements in analytical techniques, such as mass spectrometry and molecular approaches, will make it possible to identify low levels of OTA in food items more quickly.
(b) The development of tests based on nanotechnology and biosensors for OTA detection can offer extremely sensitive and selective detection techniques, obviating the requirement for sophisticated laboratory apparatus and knowledgeable staff.
(c) Predictive modelling, risk assessment, and early warning systems can be improved by incorporating machine learning and artificial intelligence algorithms into OTA analysis. These technologies can assist in locating high-risk areas and products, enabling focused monitoring and control efforts.
(d) Researchers should explore diverse avenues to combat OTA contamination. Bioengineered microorganisms or molecular modified enzymes, with optimized OTA-degrading capabilities and stability, offer promise. In fact, it is desirable to obtain new, genetically engineered enzymes with higher pH stability, active over a wider temperature range, stable over time, and characterized by high expression/high yield in microbial culture; this could be a relevant step forward. Incorporating such microorganisms, especially enzymes, into food and beverage fermentation processes could reduce contamination, especially in items like wine, beer, and dairy products.
(e) Crop susceptibility to OTA-producing molds can be decreased by developing best practices for crop management, including breeding programs for OTA-resistant crop varieties. Additionally, crop cultivation may be optimized and fungal contamination minimized with the aid of precision agriculture and weather forecasts.
(f) OTA contamination during food production and distribution can be reduced due to advancements in food processing and storage technologies. Products shelf lives can be increased while OTA levels are decreased using better packing materials, altered atmospheres, and irradiation procedures.
(g) Increased international collaboration and unified rules are also necessary. International standards, stricter laws, and the introduction of monitoring systems to verify compliance across borders are all possibilities for the future.
(h) Demand for safer products may rise with increasing customer knowledge of the dangers of OTA-contaminated food. Future applications might make use of QR codes and mobile apps to inform customers about the OTA (low) content of the goods they buy.
(i) Enhancing food safety measures in emerging markets might contribute significantly to both OTA contamination and control efforts as the world food trade expands.
(j) Individuals may have the opportunity to assess their susceptibility to mycotoxin exposure through advancements in personalized nutrition and healthcare. This information could inform dietary choices and lead to customized recommendations for minimizing OTA intake.

## 8. Conclusions

Given OTA classification as a food contaminant and its potential to impact human and animal health adversely, the imperative for developing precise and sensitive detection methods has taken precedence. Prior to quantitatively assessing OTA, samples must undergo meticulous cleansing and extraction procedures. OTA holds a prominent position among mycotoxins and is notorious for its association with food security concerns. Its global significance stems from both its toxicity and the resulting implications for human and animal well-being.

Addressing the challenges posed by OTA involves incorporating preventive measures before, during, and after harvest, alongside implementing detoxification strategies with natural, synthetic products and standardized microorganisms as the techniques to be applied. By doing so, the potential for highly toxic food products can be mitigated, thereby averting substantial challenges in the realms of food marketing, distribution, and consumption.

## Figures and Tables

**Table 1:** *Aspergillus* producing Ochratoxin A.

Organism	Section	References
*Aspergillus westerdijkiae*	*Circumdati*	[35]
*A. melleus*	*Circumdati*	
*A. ochraceus*	*Circumdati*	
*A. steynii*	*Circumdati*	
*A. subramanianii*	*Circumdati*	
*A. sesamicola*	*Circumdati*	
*A. affinis*	*Circumdati*	[36]
*A. muricatus*	*Circumdati*	
*A. occultus*	*Circumdati*	
*A. ochraceopetaliformis*	*Circumdati*	
*A. flocculosus*	*Circumdati*	
*A. pseudoelegans*	*Circumdati*	[37]
*A. roseoglobulosus*	*Circumdati*	
*A. sclerotiorum*	*Circumdati*	[35]
*A. persii*	*Circumdati*	
*A. salwaensis*	*Circumdati*	
*A. carbonarius*	*Nigri*	[38,39]
*A. niger*	*Nigri*	
*A. lacticoffeatus*	*Nigri*	
*A. sclerotioniger*	*Nigri*	
*A. alliaceus*	*Flavi*	[40]
*A. avenaceus*	*Flavi*	
*A. bertholletius*	*Flavi*	
*A. coremiiformis*	*Flavi*	
*A. leporis*	*Flavi*	[41,42,43,44]
*A. nomius*	*Flavi*	
*A. tamarii*	*Flavi*	
*A. pseudotamarii*	*Flavi*	
*A. vandermerwei*	*Flavi*	
*A. neoalliaceus*	*Flavi*	

**Table 2:** OTA occurrence in food products.

Matrix	Country	Number of Sample	Occurrence (%)	Maximum µg/kg	Mean µg/kg	Ref.
Corn, wheat	Pakistan	40	27.5	360	/	[77]
Rice	Iran	65	4.6	11.5	5.02	[78]
Barley and wheat	USA	262	12.2	185.2	/	[79]
Corn, rice, wheat, and oat-based foods	USA	489	41	9.3	/	[80]
Wheat	Canada	232	2.2	/	14.7	[81]
Wheat grain clumps around or under manhole openings of storage bins	Canada	5	100	370		[81]
Wheat and derived samples	Algeria	81	76.54	34.75	/	[82]
Cereal-based foods	Algeria	16.9	50	/	0.15	[83]
Rye	Poland	60	3	2.75	/	[84]
Fermented coffees	Brazil	14	21.4	0.87	0.18	[85]
Roasted and instant coffee	Czech	103	80.6	12.8	/	[86]
Coffee bean roasted coffee and soluble coffee	Argentina	51	69	20.3	/	[87]
Roasted and instant coffee	Chile	63	33	7.25	1.3	[88]
Roasted coffee	Spain	72	48.6	4.21	2.17	[89]
Cocoa bean	Brazil	123	22.8	7.2	1.2	[90]
Cocoa and chocolate	Canada	60	100	7.8	0.95	[91]
Dried figs	Turkey	100	8	1.72	0.64	[92]
Dried grape	Iran	66	40.9	8.4	2.98	[93]
Palm dates	Tunisia and Algeria	27	11	6.07	58.7	[94]
Raisin	USA	40	93	11.4	0.7	[95]
Dried fruit and nuts	China	253	1.6	9.39	6.23	[96]
Chili sauce	Pakistan	252	71	114	/	[97]
Allspice, pepper, chili, cinnamon, ginger, and mixture	Italy	94	30	34	7.1	[98]
Grounded sweet pepper	Italy	8	100	/	23.6	[99]
Dried sweet pepper	Italy	23	39	/	53.9	[99]
Salami	Italy	50	10	103.69	/	[100]
Dry-cured ham	Italy	110	76.4	5.64	/	[101]
Dry-cured ham	Italy	18	22.2	69.3	/	[102]
Cheese	Italy	30	13.3	4.7	/	[102]
Cheese	Italy	84	8.3	22.4	/	[20]
Beef burger	Egypt	25	100	/	4.55	[103]
Chicken meat	India	115	41	/	1.41	[104]
Eggs	India	80	35	/	1.17	[104]
Milk	China	120	25.8	18.8	/	[105]
Red wine	Croatia	110	98.2	0.163	0.040	[106]
Red, rose, and white wine	Serbia	113	52.2	0.134	0.026	[107]
Wine	Spain	40	47	2.28	1.13	[108]
Beer	Mainly Portugal	85	10.6	11.25	/	[85]

**Table 3:** OTA maximum levels permitted in different countries for main food matrices.

OTA Maximum Level in Different Countries (μg/kg)							
Matrix	European Union	Brazil	China	Turkey	Egypt	Vietnam	Codex Alimentarius
Dried vine fruits (currants, raisins and sultanas) and dried figs	8.0	10.0		10.0	10.0	3.0	
Other dried fruits	2.0	10.0					
Dried herbs	10.0						
Sunflower seeds, pumpkin seeds, (water) melon seeds, hempseeds, soybeans	5.0						
Unprocessed cereal grains	5.0	10.0	5.0	5.0	5.0	5.0	5
Bakery wares, cereal snacks and breakfast cereals	2.0			3.0	3.0	3.0	
Roasted coffee beans and ground roasted coffee	3.0	10.0	5.0	5.0	5.0	5.0	
Soluble coffee (instant coffee)	5.0	10.0	10.0	10.0		10.0	
Cocoa powder	3.0						
Cocoa beans		10.0					
Dried spices	15.0			15.0			
*Capsicum* spp. (dried fruits including chillies, chilli powder, cayenne or paprika)	20.0	30.0		30.0			20.0
Wine, fruit wine, aromatized wine, grape juice, grape must	2.0	2.0	2.0	2.0		2.0	
Baby food and processed cereal-based food, for infants and young children	0.50	2.0		0.50		0.5	

**Table 4:** Analytical methods for OTA determination.

Methods	Matrix	LOD *	LOQ *	Ref.
LC-MS	Cheese	0.05 µg/kg	0.15 µg/kg	[143]
LC-MS/MS	Bovine meat	0.059–291.36 μg/kg	0.081–328.13 μg/kg	[144]
2D-HPLC	Beer, wine, corn, coffee	21.2 pg/mL	64.3 pg/mL	[145]
HPLC-MS/MS	Maize	0.26 μg/kg	0.87 μg/kg	[146]
UHPLC-MS/MS	Coffee	3 pg/g	1 pg/g	[121]
HPLC-fluorescence detection (FLD)	Spices	0.03 ng/g	1.0 ng/g	[147]
LC- LC-MS/MS	Cereals	1 pg/kg	_	[148]
Nanofluid extraction coupled with HPLC with fluorescence detector HPLC–FLD	Food	0.2 μg/kg	0.5 μg/kg	[122]
SPE–HPLC	Wine	0.03 µg/L	0.10 µg/L	[149]
qPCR	Coffee	3.85 × 10^3^ copies of the PKS gene	_	[150]
magnetic beads (MBS) ELISA	Cereal	0.07 ng/mL	0.249–5.28 ng/mL	[151]
magnetic solid-phase extraction (MSPE) GC-MS/MS	Beer, Chinese Baijiu and vinegar	0.03 µg/L	0.1–800 μg/L	[152]
ELISA	Cereals	0.001 ng/mL	0.003–0.673 ng/mL	[142]
Electrochemical aptamer sensors	Food	6.5 µM	_	[153]
Photoluminescence immunosensor	Food commodities	0.01 ng/mL	_	[154]
Nanobody (Nb) Förster Resonance Energy Transfer (FRET) immunosensor	Cereal	5 pg/mL	_	[155]
Electrochemical (EC) sensors	Food	0.38 ng/mL	1–20 ng/mL	[156]
Aptamer based sensors	Food	0.88 pg/mL	1 pg to 300 ng/mL	[157]
Fluonanobodies (FN) nanosensor	Cereals	5 pg/mL	5–5000 pg/mL	[10]
Quartz crystal microbalance with dissipation monitoring (QCM-D)	Red wine	0.16 ng/mL	0.55 ng/mL	[158]
FRET-LFI	Coffee	0.88 ng/mL	_	[159]

* LOD: Limit of detection; LOQ: Limit of quantification.

**Table 5:** Some chemical methods employed for OTA degradation.

Method	Agents	Condition	Food Matrix	Effect on OTA	Ref.
Alkalinization	Potassium carbonate	40 °C for pH 10	Grape	Reduced by up to 50%	[184]
	Ammonia	High pressure (60 psi) and normal temperature	Wheat	Reduced by 79%	[185]
	Potassium carbonate	2% solution at 90 °C and 6894.76 kPa for 10 min.	Cocoa shells	Reduced by 93%	[186]
Acidification	Acetic acid, citric acid, lactic acid, or hydrochloric acid	50 °C for 24 h at pH 2	Grape pomaces	Reduced by up to 67.23% (lactic acid)	[187]
Ozonation	ozone	100 mg/L for 180 min	Corn	Reduced by 70.7%	[188]
	ozone	gaseous ozone at 12.8 mg/L for 240 min	Sultanas	Reduced by 82.5%	[189]

**Table 6:** Degradation of OTA by physical methods in food.

Method	Condition	Food Matrix	Effect	Ref.
Heat treatment	120 °C and 180 °C for 30 min and 60 min	Oats	Reduction of 2–18%	[198]
	150 °C for 50 min	Pistachios	Reduction of over 60%	[199]
Ultraviolet radiation	180 min of UV radiation	Poultry feed	Completely decontaminated	[200]
Gamma radiation	20 kGy,	Corn	Reduction of 61.1%	[201]
	30.5 kGy	Wheat flour, Grape juice, and wine	Reduction of 24%, 12%, and 23%, respectively	[202]

**Table 7:** Elimination of OTA through adsorption.

Material	Adsorbents	Medium/Matrix	Removal	Ref.
	Ground nuts, coconut fiber, waste coffee grounds, and citrus peel	Liquid (ethanol/water mixture = 14/86, *v*/*v*)	Up to 100%	[233]
Natural	Powdered *Pleurotus ostreatus*	in vitro gastrointestinal digestion	85% at the end of the intestinal phase	[227]
	Egg albumin, gelatin, chitin, and chitosan	Red Wine	Between 13% and 34%	[234]
Inorganic Mineral	Activated carbon 5 mg/mL	In vitro	More than 89%	[226]
	Activated carbon	PBS and wine	87–100%	[228]
	Activated carbon	Red and white wine	Up to 100%	[230]
	Bentonite-orange peel extract	Gastrointestinal fluids	Up to 2.13 mg/g	[235]
Organic Synthetic	Cyclodextrin-polyurethane polymer	Wine Samples	Up to 10 μg/L	[236]
	Clay polymer nanocomposite	grape juice and wine	Up to 92% (15 μg/L)	[237]

**Table 8:** OTA control by microorganisms and enzymes.

Microorganism/Enzymes	Source	Reaction Condition	OTA (μg/mL)	Degradation Rate (%)	Ref.
Bacteria/Actinobacter					
*Rhodococcus erythropolis* GD2A, BRB 1AB	Natural soil	72 h/liquid medium	2	27–34	[246]
*Cupriavidus basilensis* ŐR16	Soil	5 d/liquid medium	100	>90	[247]
*Brevundimonas vesicularis*	Vineyard soil	28 °C/water	1	100	[248]
*Acinetobacter calcoaceticus* strain 396.1	Vineyard soil	6 d/liquid medium	1	82	[249]
*Acinetobacter* sp. Neg1	Vineyard soil	6 d/liquid medium	1	>70	[250]
*Bacillus amyloliquefaciens* ASAG1 a	Grain, maize	31 °C, 10 h/nutrient culture media	1	98.5	[176]
*Luteimonas* sp. CW574	Soil and moldy food	48 h/feed	0.02	48.3	[251]
*Alcaligenes faecalis* ASAGF 0D-1	Soil	48 h/liquid medium	1	92	[252]
*Streptomyces* AT10, AT8, SN7, MS1, ML5, G10,PT1	Soil	5 d/liquid medium	0.095	23–53	[253]
*Bifidobacterium bifidum* CECT 870T, *B. breve* CECT 4839T; *Lactobacillus casei* CECT 475T, *Lactobacillus casei* CECT 4040, *L. casei* CECT 4045, *L. delbrueckii bulgaricus* CECT 4005, *L. johnsonii* CECT 289, *L. paracasei* CECT 4022, *L. plantarum* CECT 220, *L. plantarum* CECT 221, *L. plantarum* CECT 222, *L. plantarum* CECT 223, *L. plantarum* CECT 748, *L. plantarum* CECT 749, *L. rhamnosus* CECT 278T, *L. rhamnosus* CECT 288, *L. salivarius* CECT 4062	Spanish Type Culture Collection	24 h/liquid medium	0.6	30–97	[254]
*Lactobacillus rhamnosu*	Winegrapes	TSB medium	1	55	[255]
*Bacillus subtilis* CW14	fresh elk	30 °C, 24 h/liquid medium	1	71.3	[256]
*Leuconostoc paracasei* ssp. *Paracasei* (3T3R1), *L. mesenteroides* ssp. *Dextranicum* (T2MM3)	Food	20, 25, 30 °C, 10 days/MRS-CYA20S medium	NR	7.3–100	[257]
*Brevibacillus* sp.(ALJ01), *Brevibacillus schisleri* (ALJ02)		24 h/MSM medium	5	89.8, 96.5	[258]
Fungi					
*A. carbonarius* SA332	French grapes	5 d/liquid medium	2	83	[259]
*Botrytis cinerea* UdLTA 3·95, UdLTA 3·102, UdLTA 3·115	Grapes	7 d/solid grape synthetic medium	1	24.2–26.7	[260]
124.*niger* M00120	Soil	2 d/liquid medium	0.2	99	[261]
*Aureobasidium pullulans* AU14-3-1, AU18-3B, AU34-2, LS30	Apple leaves, Plum fruits, Grapevine leaves, Apple	6 d/liquid medium	0.8	75–90.5	[262]
*A. carbonarius* 10614, *A. niger* 10,443	Wine grapes	25 °C, 7 d/Wine	6	83.44	[263]
Yeast					
*Yarrowia lipolytica Y-2*	Vineyard soil	2 d/liquid medium	1	84	[249]
*Yarrowia lipolytica*	vineyard	2 d/liquid medium	1	88	[264]
*Yarrowia lipolytica* Y-2	The surface of grapes	28 °C/liquid medium	1	97.2	[264]
*Metschnikowia pulcherrima* MACH1, M320; *Kloeckera lindneri* GAL5; *Pichia guilliermondii* M8, M29; *Rhodococcus erythropolis* AR14	Agroinnova culture collection centre (Italy)	15 d/liquid medium	7.5	25.8–84	[265]
Enzyme					
Carboxypeptidase	*Bacillus amyloliquefaciens*	Overnight/Tris-HCL	10	72	[177]
Hydrolase	*Aspergillus niger* MUM 03	4 h/phosphate buffer	1000	Up to 154 ng/min g	[266]
Amidase 2	*Aspergillus niger*	30 min/Mops-NaOH	0.85	83	[267]
Carboxypeptidase PJ_1540	*Acinetobacter* sp. *Neg1*	Overnight/Tris buffer	1	33	[268]
Carboxypeptidase cp4	*Lysobacter* sp. CW239	24 h	250	86.2	[269]
Peroxidase	*Armoracia rusticana*	72 h/PBS	0.01	27	[270]
Laccase	*Pleurotus eryngii*	72 h/sodium acetate buffer	0.05	27	[271]
Carboxypeptidase	*Bacillus subtilis*	48 h/PBS	1	71.3	[256]
Metalloendopeptidase	*Bacillus subtilis*	1 h, 41 °C/Phosphate buffer pH 4.6, 5 and 7	0.1	between 8.2 and 45.3%	[272]
Amidohydrolase ADH3	*Stenotrophomonas acidaminiphila* CW117	24 h, 60 °C/PBS, pH 7.2	0.05	100	[273]

## Data Availability

No new data were created or analyzed in this study. Data sharing is not applicable to this article.

## Supplementary Materials

The following supporting information can be downloaded at: https://www.mdpi.com/article/10.3390/foods13081184/s1, Table S1. Main mycotoxins, fungal producing species, major contaminated foodstuffs, and their effects on humans and animals [11,12,13,14].
