# Supplementary material for: Comprehensive Insights into Ochratoxin A: Occurrence, Analysis, and Control Strategies
Source: Foods. 2024 Apr 12;13(8):1184. doi: 10.3390/foods13081184 (PMC11049263; doi:10.3390/foods13081184)
Supplement: Supplementary file 1 [file foods-13-01184-s001.zip › foods-2929972-supplementary.pdf]

Table S1. Main mycotoxins, fungal producing species, major contaminated foodstuffs, and their effects on humans and animals [11-14].

Mycotoxins	Fungal species	Contaminated foodstuffs	Toxic effects
AFs	*A. flavus, A. parasiticus, A. Nomius, Penicilium*	cereals, fruits, dried fruits, milk	Carcinogenicity, hepatotoxicity, teratogenicity, suppression of immune systems, alteration of DNA structure, hepatitis, hemorrhage, renal lesions
Trichothecenes (DON, T2, HT2…)	*F. graminrarum, F. culmorum, F. sporotrichioides*	Cereals and legumes	Induction of apoptosis in progenitors, hematopoietic tissue and immune cells, impact on protein synthesis, alteration of immunoglobulins
OTA	*A. ochraceus, P. nordicum, P. verrucosum, A. carbonarius, A. niger, A. Alliaceus, A. terreus*	Wine, grapes, coffee, cocoa, Cereals	Kidney and liver damage, loss of appetite, nausea and vomiting, immune system suppression, carcinogenic
FB	*F. moniliforme, F. verticillioides, F. Proliferatum*	Cereals (corn)	Carcinogenic, neurogenic, liver damage
Patulin	*A. cyclopium, P. expansum, P. granulatum, Paecilomyces variorti*	Cereals and fruits	Brain hemorrhage, neural disorders, skin lesions, skin cancer, lung, mutagenicity, antibacterial impact
ZEA	*F. graminearum, F. culmorum, F. semitectum*	Cereals	Carcinogenic, hormonal imbalance estrogenic effect, reproductive problems, teratogenic
